# A personal view on basic education in reproduction: Where are we now and where are we going?

**DOI:** 10.1111/rda.13769

**Published:** 2020-09-24

**Authors:** F. Herman Jonker

**Affiliations:** ^1^ Department of Population Health Sciences Section Farm Animal Health Faculty of Veterinary Medicine Utrecht University Utrecht The Netherlands

**Keywords:** course design, education, interactive e‐learning, skills laboratories, teaching reproduction

## Abstract

This article explores the current and expected direction of education in reproduction at the Faculty of Veterinary Medicine of Utrecht University. The current reproductive course in the Bachelor's programme is described. Based on the yearly routine course evaluation, changes have been started and continue to be implemented, and the educational ideas behind it are defined. Interactive e‐learning modules that combine knowledge clips, animations, and quizzes have been developed. For the practical classes, e‐modules with instructional videos have been paired to the written material. Using these new tools during self‐study, students have to prepare for the necessary face‐to‐face classes that contain more in‐depth discussions and practical training. In the second part, the author describes his expectations for further educational development. The growth of effective self‐study using e‐learning, besides traditional textbooks, before more in‐depth face‐to‐face classes is likely to occur. With the growth of modern possibilities, such as the haptic technique and virtual reality, a better preparation in laboratory skills before practical training with animals is expected. In the author's opinion, despite all new learning methods and material, small group, face‐to‐face lectures, and practical classes with animals or animal material remain absolutely necessary. This article concludes with some lessons learned during the current adaptation of the course.

## 1 CURRENT CURRICULUM

The current curriculum started in 2007 when the Faculty of Veterinary Medicine of Utrecht University (FVMU) adopted the Bachelor's/Master's curriculum. The Bachelor's and Master's programmes are separate and have different learning objectives. Currently, the Bachelor's degree is a prerequisite to start the Master of Veterinary Medicine programme. At the end of the 3‐year Bachelor's programme, the student has knowledge and insight in normal anatomy and physiological and pathophysiological processes; is aware of diagnostic methods and the possibilities of intervention for individual and groups of animals and can use these results in clinical reasoning; and can prepare a therapy and intervention plan. Besides these veterinary competencies, the student also has knowledge and insight in legal, ethical, and One Health aspects; has an academic attitude and solves problems with evidence‐based solutions; can communicate clearly and effectively; is capable of effective and multidisciplinary cooperation; and reflects on and takes responsibility for her or his professional actions (Kremer & Haeften, 2017). The Bachelor's degree is a pre‐clinical programme and is organised around specific themes. The first year covers the basic structures and processes of the body, with a specific interest in cells and molecular processes. The second and third years deal with more organ system–orientated courses; for instance, courses on respiration and circulation, digestion, and reproduction. They cover topics from healthy, normal physiology to disease states. Normal anatomy, histology, physiology, pathophysiology, and pathology are integrated into one course. This organisation is in contrast to the previous curriculum that utilised separate physiology and pathophysiology courses, besides the organ system courses. Compared to the previous curriculum, in the Bachelor's programme, the focus is on small group seminars besides traditional lectures. The latter only present the overview and the important facts and their number has been significantly decreased. The seminars have a maximum of 25 students; they comprise questions or cases that are discussed in the group. Seminars are prepared by students in advance using their textbooks and syllabi. Active discussions and interaction with peers on the specific topics or cases in these seminars promote active learning and should help the student to more deeply learn and retain knowledge (Bouwmeester et al., 2019; Hodgson & Ilkiw, 2017; Spruijt, Jaarsma, Wolfhagen, van Beuklen, & Scherpbier, 2012). The seminars can be seen as a form of flipping the classroom. Prerequisite in this type of education is a good preparation of the students, enabling active and more in‐depth discussions and application of the acquired knowledge. During these seminars, students should be in the lead. The role of the teacher is more supportive: to stimulate and direct the discussion.

The three‐year Master's programme is clinically oriented and results in the DVM degree. The graduate is a qualified veterinary general practitioner and also has qualifications corresponding with her or his chosen Master's programme. The graduate has followed advanced training in specialist areas of veterinary science according to personal interests and has relevant knowledge and expertise to perform preventive, diagnostic, medical, and surgical procedures for the health, welfare, and treatment of animals, appropriate to the context and life stage of the animal. The graduate designs and implements programmes to improve the health, welfare, and productivity of animal populations. Besides the veterinary competencies, the graduate also has knowledge and insight in legal, ethical, and One Health aspects; has an academic attitude and solves problems with evidence‐based methods; can communicate clearly and effectively; is capable of effective and multidisciplinary cooperation; and reflects on and takes responsibility for her or his professional actions (Benedictus et al., 2018). The veterinary Master's programme consists of clinical rotations combined with specific theoretical lessons; these are frequently presented in seminar form. Students choose Companion Animal, Equine, or Farm Animal Health/Veterinary Public Health as their main direction, but they also rotate in the other two areas. In the Farm Animal Health track, specific attention is given to herd analysis based on herd data combined with farm, herd and animal inspection. During the rotations, students are evaluated on theoretical knowledge and practical skills as well as the seven competencies of the Vetpro framework: Veterinary expertise, Communication, Collaboration, Entrepreneurship, Health & Welfare, Scholarship, and Personal development (Bok, Jaarsma, Teunissen, van der Vleuten, & van Beukelen, 2011). The FVMU Master's programme is organised as competency‐based education (CBE). The development of the students in the seven competencies during their education is monitored and assessed with an electronic portfolio. This CBE and the programmatic assessments (PA) provide the student with regular feedback on her or his position in relation to the end goals of the curriculum for different competencies. The students can follow their own development and compare this with the average of their peers. Hence, students are given insight in their strengths and weaknesses in each competency and determine where further education is needed. In the student's personal development plan, which is part of the portfolio, she or he describes her or his specific educational plans. A formative portfolio assessment is done twice a year by the tutor. At the end of the second and third years, there is a summative assessment (Bok, 2015).

Entrustable Professional Activities (EPAs) are a further development in CBE (Bok & Jaarsma, 2017; Duijn, ten Cate, Kremer, & Bok, 2019). They indicate the level that has already been reached on (often clinical) tasks in relation to the desired day‐one competency level. In this way, EPAs also allow a more personalised, flexible progression of the study. At FVMU, EPAs for the different veterinary masters are currently being developed.

In the author's opinion, CBE, PA, and EPAs are important parts of the total Master's education programme. They provide meaningful feedback to the student during their study progress and are crucial to monitor and assess learning progress and steer self‐directed learning (Favier, ten Cate, Duijn, & Bok, 2020). Given that CBE, PA, and EPAs are more monitoring and steering instruments, they will not be further addressed in this paper.

## 2 EDUCATING REPRODUCTION AT FVMU FROM 2010 TO 2015

The seven‐week reproduction course at the FVMU is positioned in the third year of the Bachelor's programme at the beginning of the first semester. Within the reproduction course, 11 themes have been formed to provide a better overview for the students (Table 1). Each theme starts with at least one traditional lecture that highlights what will be included in the theme, followed by one or more seminars and practical classes. Similar to all other Bachelor's courses, the students must attain a lot of knowledge. In practice, a large proportion of the students in the seminars remained passive learners, ‘only’ waiting for the correct answers or solutions to the questions and cases. Further applying and deepening knowledge was insufficient. Although examination results were good, the retention of the knowledge after a certain period of time when students entered the Master's programme was poor.

**Table 1:** Thematic chapters of the Faculty of Veterinary Medicine of Utrecht University (FVMU) Bachelor's programme course in reproduction

	Theme	Subject
	Introduction	General introduction and outline of the course
1.	Anatomy and development	Male and female reproductive tract
2.	Oestrus Cycle	Oestus cycle and regulation, puberty, anoestrus, and disturbances
3.	Spermatogenesis	Spermatogenesis, ejaculation, and abnormalities
4.	Fertilisation	Fertilisation and placentation
5.	Pregnancy	Pregnancy, abnormalities, and abortion
6.	Parturition/dystocia	Normal parturition and dystocia
7.	Puerperium	Puerperium
8.	Neonatology	Perinatology, the first days of the neonate
9.	Mammae	Mammae, lactation, and mastitis
10.	The practice	Abnormalities in physiology and anatomy
11.	Bird reproduction	Reproduction of birds

In the regular yearly student evaluations for each course in the Bachelor's programme, our reproduction course was well appreciated and received good scores. The general appreciation was around 7.5 on a 10‐point scale, and around 75% of the students passed their exam at the first attempt. However, for years the general complaint was the high workload in this seven‐week course. Students indicated they often had a lack of preparation time for their classes, partly explaining the poor preparation for seminars and practical classes. A high workload is strongly related to a surface approach, lower grades, and, consequently, a lower retention rate (Ryan, Irwin, Bannon, Mulholland, & Baird, 2004). This high workload is a more general concern among veterinary and medical students (Collins & Foote, 2005; Killinger, Flanagan, Castine, & Howard, 2017).

A partial explanation for the high study load in the reproduction course is the fact that, for good comprehension of reproduction, almost every species has to be studied separately – due to differences in reproductive physiology. For the theriogenologist, this is particularly interesting and stimulating. However, these differences are confusing and puzzling for the student. In addition, to be as complete and accurate as possible, there is a significant amount of knowledge about reproduction and its regulation that must be learned. Hence, there is a high study load in a relatively short period and, consequently, a lack of deep learning (Bouwmeester, de Kleijn, ten Cate, van Rijen, & Westerveld, 2016; Grant, 2014). The result was that more students came to class low prepared and remained more passive learners. Later, in their Master's programme, retention of knowledge, including basic physiological principles, was low. Instead of further extending reproduction knowledge in more practical cases, the theory from the Bachelor's programme had to first be refined.

## 3 CHANGES IN THE REPRODUCTION COURSE

In 2015, the Bachelor's reproduction course was evaluated by a group of teachers and students involved in the course; they provided clear points for improvement. Like in other studies (Malone, Root Kustritz, Rendahl, & Molgaard, 2020), during this evaluation the students provided valuable input and remained involved in the process. The participants highlighted that our education was still too teacher oriented and contained a lot of material. During this evaluation, the participants highlighted that students nowadays are much more technology‐minded and are adapted to use more electronic devices instead of textbooks (Gledhill, Dale, Powney, Gaitskell‐Phillips, & Short, 2017; Senger, Oki, & McLean, 2012). As one of the first courses in the FVMU curriculum, we therefore decided to redesign our course in a more blended way by implementing more e‐learning and flipping the classroom. We expected this change would benefit both students and faculty, as recently confirmed by Matthew, Schoenfeld, Danielson, and Warman (2019). During this reform, we modified neither the curriculum itself nor the learning objectives of the course. Blended learning – a mix of e‐learning and traditional classroom learning – had already been used in the preparation of the seminars, but it has been enhanced. E‐learning includes all digital learning material that is offered through an electronic route. It is often web‐based and has, in general, a positive impact on students’ learning (Gormley, Collins, Boohan, Bickle, & Stevenson, 2009). Amongst others, examples of e‐learning material are video‐recorded lectures; instructional videos and videos of clinical cases; digital microscopy; virtual patients; and interactive modules. In our renewed course, e‐learning has been significantly extended. Many of our recorded lectures and several videos had already been made available through the electronic learning management system (LMS). After class, students could recall the lecture and study any parts that remained unclear. These videos mainly gave information on parturition processes of several species or showed clinical examinations. These recorded lectures comprised narrative slides, which are helpful and lead to better examination results (Bouwmeester et al., 2016; Chen & Lin, 2012). Moreover, in our reproduction course we noticed a significant decrease in lecture attendance. In a short survey, our students indicated that because these lectures were available on the LMS, it was more convenient to listen to them at home at a convenient time for them. Some of the students indicated that they only listened to the lecture, displaying a passive learning style, whereas others showed active learning: they stopped the lecture to look up unclear parts in the textbook before continuing. Based on this short survey – part of the regular course evaluation – we decided to skip some of the live lectures and only provide these as recorded forms through the LMS. This change has created more free study time for our students and has also decreased the contact time for teachers. In the near future, more lectures will only be presented online. A point of attention will be to keep these e‐lectures updated.

## 4 CHANGING THE THEORETICAL EDUCATION FORM OF THE COURSE

**Figure 1:**
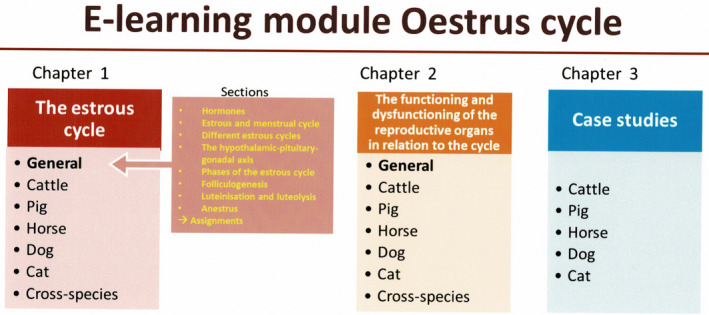
Main menu structure of the e‐learning module of the oestrus cycle. The three chapters contain several paragraphs with subsections. When browsing the menu, the section structure pops up and a section can be started

As a major change in our course, and to stimulate active learning, we decided to develop new, more attractive, and inspiring e‐learning. E‐learning is complementary to the textbook and should be available through the LMS so that it can also be reviewed at home during convenient self‐study times. We developed the first interactive e‐learning module, on the oestrus cycle, with a mixture of animations, text, pictures, movies showing behaviour or clinical examination instruction, knowledge clips, and quizzes with immediate feedback (Jonker et al., 2017). It took about a year to make this module; we were luckily able to appoint one person for about 50% of the time to build the module. The module comprises several chapters and paragraphs that can be started through the main menu (Figure 1). Once a section is started, the student has to follow the whole section, to avoid ‘cherry picking’. We have included several animations to elucidate dynamic, complicated processes. Animations and film clips in the module provide more variation in e‐learning and trigger both visual and auditory memory (Noyes, Carbonneau, Gotch, & Matthew, 2020). Animations can also simplify more complex descriptions – for instance, regulatory feedback mechanisms – and support the comprehension and application of knowledge (Hwang, Tam, Lam, & Lam, 2012; Upson‐Taboas, Montoya, & O’Loughlin, 2019). Quizzes and testing benefit learning because the learner has to actively recall the previously learned information (Augustin, 2014; de Kleijn, Bouwmeester, Ritzen, Ramaekers, & van Rijen, 2013; Riemer & Schrader, 2015). In the case of a repeated incorrect answers, the student is referred to the corresponding part of the module and the relevant chapter in the textbook. In the yearly course survey, our module has been highly appreciated by the students; they have indicated the module significantly affects their understanding of oestrus cycle physiology in the different species (Jonker et al., 2017). Students have indicated that they would use the module again in their Master's programme and asked for more e‐learning modules on several subjects, as well as for other courses. At the moment, more e‐learning modules on other reproductive topics are being made: one on spermatogenesis is almost ready.

In the new course concept, the face‐to‐face seminar classes have changed. As a result, there has been a reduction in their number and an adaption of the content and form of the remaining ones. Redundant seminars have been removed and some seminars have been made shorter, but more interactive and intensive. Together with the reduction in traditional lectures, more self‐study time has been created for students and less time is required for teaching staff to moderate the seminars. Through the increased self‐study time, we hope to reduce the student's workload and increase the possibility of knowledge retrieval, which benefits learning outcomes (Dobson, 2012). Similar changes in learning methods are now being made in other discipline‐specific courses.

To obtain an optimal effect, students should be well instructed on the purpose of the new education method. In the new setting, they should be more focussed on clarification of problems encountered during the homework and applying knowledge. In the author's opinion, cases used in these seminars should be meant to apply the knowledge and the underlying concepts. In this way, the student has to use the just‐acquired knowledge, a phenomenon that will allow in‐depth learning (Bouwmeester et al., 2019) and clarify the concept(s) related to this subject. Solutions for specific clinical cases can then be dealt with in the master's programme.

## 5 THE FUTURE OF THEORETICAL COURSE CONTENT

In the author's opinion, when changing or building a course, it is important to carefully think about the content in relation to the final goal of the course and the students’ workload. Hence, before starting the course construction, the question ‘Do we need to teach everything?’ must be answered. In recent years, the author has come to the conclusion that ‘less is more’, especially in the Bachelor's programme phase of the study. This outlook is in line with the advice from the study of Cavalieri (2009) that colleges should be aware of the study load for their students and avoid too much pressure on the students, an eventuality that might lead to superficial learning. Instead of already dealing with the majority of pathological conditions, in the Bachelor's programme, the focus should be on the important knowledge and concepts, including species differences. In this phase, clinical cases should be supportive and illustrative. Later, in the Master's programme, knowledge and concepts from the Bachelor's programme will allow important cases for day‐one competency to be taught. In general, teachers have the difficult task to discuss which specific pathological conditions are to be in the new course and what can be omitted from the current material and be offered later. These specific examples should support the concepts and principles. The critical question should be: does this contribute to a better understanding? Of course, the overall aims of the course and curriculum must be kept in mind and lead these decisions. As the lifelong learning concept is generally accepted, students with adequate knowledge of the main concepts and principles are expected to easily acquire reliable additional information for new cases (May & Silva‐Fletcher, 2015).

## 6 TRAINING CLINICAL SKILLS

Besides theoretical knowledge, students are also taught clinical skills, like clinical examination techniques, obstetrical procedures, and, more in the Master's programme, internal rectal exploration. This method has been traditionally learned through books and lectures, followed by a practical training. The actual training with animal material or live animals always remains necessary, but good preparation is essential to overcome welfare issues as much as possible (Ghosh, 2017). Instructional films of clinical examination procedures and surgical techniques are a useful aid in preparing students for the actual clinical training, as well as simple hands‐on exercises in a skills laboratory (Jang & Kim, 2014; Klupiec et al., 2014; Müller, Tipold, Ehlers, & Schaper, 2019). Instructional films are easily available through the LMS, and they can also be accessed on mobile devices. Easy access allows the student to be prepared for the actual training and provides more and better training opportunities (Kelly, Mihm‐Carmichael, & Hammond, 2019). Better preparation during self‐study enhances student‐led learning (Kelly et al., 2019) and will make practical classes more efficient – and possibly shorter. In the future, perhaps admission to classes using animals should only be allowed after introductory material is completed through the LMS. Easy access might also be helpful as a quick refresher; for instance, when in clinical rotations, a student can assist in a clinical examination or surgical procedure. Besides easy access, the format of the material must be adapted to the possible connection speed (Jang & Kim, 2014). We also found this appreciation of video instruction by the students in our study; students have already valued a preliminary e‐learning module on correcting abnormal positions with a film, without narrative comment, in their preparation for the practical lesson (Jonker, Scholten, van der Veen, Joosten, & Jansen, 2019). The design of this module on vaginal deliveries allows quick switching among subjects.

Using these instructional videos does not mean that written text is redundant. Narrative comments in the video are brief and supportive for the pictures. The written text can provide more detailed information and explanation. The video and text should be complementary. Based on differences in learning styles, some students recall the video they previously viewed, but other students recall their written notes made during lectures or reading textbooks (Jang & Kim, 2014; Langebæck, Tanggaard, & Berendt, 2016) Using this module along with the written text makes the extensive instruction lecture before the start of each practical class redundant; hence, the class is more efficient (Kelly et al., 2019). It is important to clearly instruct students that preparation is essential (Bouwmeester et al., 2016). Having the videos available in a practical room is favourable as a reference possibility for the student. The number of instructional videos will likely further increase and can be added to modules with a clear and easy‐to‐navigate structure.

## 7 TRAINING EXAMINATION OF THE INTERNAL REPRODUCTIVE ORGANS

Training for internal (reproductive) examinations and techniques has a special place. It has always been a relatively big step between the textbook pictures and slaughterhouse material and a living animal. Guiding a student through their first steps in internal examination is difficult because the teacher cannot control what the student is palpating. Consequently, when students start transrectal palpations for reproductive examinations, it often takes several training sessions before the reproductive organs can be properly identified (Bossaert et al., 2009). However, the high cost of keeping teaching animals often leads to a large student‐to‐animal ratio, a phenomenon that makes it more difficult for students to gain sufficient experience (Crossan, Brewster, & Mellor, 2001). Even when teaching is offered in slaughterhouses, the number of available animals is scarce (Lopes & Rocha, 2006) and the circumstances might be less safe. Moreover, ethical considerations limit a student's opportunities to gain experience because rectal palpation is a stressful procedure and animals need a period of rest after being used for training. Animals used for teaching are considered experimental and their use should be minimised as much as possible, implementing the 3Rs: reduction, replacement, and refinement of animal experiments.

Therefore, training for internal examinations and techniques in a skills lab is a good and valuable exercise for students to gain confidence before going to a living animal. For obstetrical techniques, like diagnosing and correcting abnormal posture, many veterinary colleges utilise spontaneous stillborn foetuses in a phantom. This method has already been used for a long time; it provides a good impression and favours the development of competence. Disadvantages are the amount of necessary material, possible risk of infection and the planning and preparation time – most foetuses are frozen after collection and will need 2–3 days out of the freezer before they can be used. Once used, they have to be discarded and cannot be frozen again. This planning can be overcome by models/mannequins that are available for use in skills laboratories. The obstetrical Holstein Dystocia Simulator (Veterinary Simulator Industries Ltd., Canada), similar to what we use at FVMU, enables recognition of the posture of the calf and correction of an abnormal posture; it also allows an assisted vaginal delivery by traction.

For rectal exploration in a skills lab there are specially mannequins like Sensa (Jonker et al., 2012), BreedingBetsy (Brad Pickford, Australia), Henryetta (MW Design, New Zealand), and the more sophisticated Haptic Cow and Haptic Horse (Virtalis, UK). Virtual reality (VR) training systems help students bridge the gap between pictures and slaughterhouse materials and real living animals (Annandale, Annandale, Fosgate, & Holm, 2018; Baillie, Crossan, Brewster, May, & Mellor, 2010; Bossaert et al., 2009). In these mannequins, the localisation of the different parts of the reproductive tract, including some abnormalities, can be trained, and in some mannequins it is also possible to practice difficult techniques, like inserting a pipet in the cervix. During training, the instructor can see what the students are doing and direct their movements. In addition, there is no limit to the number of students who can be trained at one time and these mannequins are instantly available. Students can train several times until they feel more confident; subsequently, they will likely perform better in a living animal. It is correct that using models in teaching practical skills lack the variations found in living animals, a factor that is sometimes seen as a disadvantage (Crossan et al., 2001). Still, by using models for training, fewer animals are needed to reach a minimal level of experience (Bossaert et al., 2009; Crossan et al., 2001; Nagel, Ille, Aurich, & Aurich, 2015). At FVMU, the use of these mannequins has been implemented in the regular training for students in the master's programme. Shortly after this training, the first training in living animals in a teaching environment is scheduled, before entering the clinic. During this first life training in a safe learning environment, students now indicate more often the recognition of the reproduction organs, an outcome increases their confidence. Mannequins could be used in examining clinical skills during a objective structured clinical examination, OSCE‐ tests, as assessment in skills lab is also possible (Baillie et al., 2010). Again, it should be emphasised that models are used in training before real living animals; hence, this technique is complementary and not a complete replacement for training in living animals.

## 8 THE AUTHOR’S EXPECTATIONS FOR UPCOMING EDUCATION

A further increase in the use of interactive e‐learning modules is likely to occur. There seems to be a change from early adopters to more regular implementation of e‐learning beyond the field of reproduction. Students are asking for more and are appreciating e‐learning; once available, it will likely improve learning outcomes. The new e‐learning modules might also experiment with using serious gaming because it has a positive effect on learning and fits the general interest of students. However, the effect on learning varies. Gaming should be used carefully because it is not appreciated by all students (Riemer & Schrader, 2015; Upson‐Taboas et al., 2019). A further extension of virtual patients and cases, although labour intensive, can be expected as a prelude for the work with real‐life cases in a teaching clinic; nevertheless, it cannot replace real‐life cases. Virtual patients are highly appreciated by the students. This modality helps them to apply previous knowledge and gives them more confidence in a clinical setting (Sawras, Khosa, Lissemore, Duffield, & Defarges, 2020). New virtual cases could also include a virtual herd analysis.

VR, either on portable devices or computers, will be further implemented because it helps to provide additional insights. Several VR‐modules are already available, for instance, anatomy (3D Canine and Cow, Virginia Tech and University Libraries), and equine obstetrics (FoalinMare, ugent.be); more can be expected to be developed.

Face‐to‐face learning at FVMU will change but remain necessary, as will practical classes. Face‐to‐face classes will be more learner orientated, with more discussions and tactics to apply the knowledge, making it more meaningful. In the Master's programme, these discussion seminars have already been applied in some courses. During self‐study time, students review recorded lectures and e‐learning, followed by a face‐to‐face seminar with more interaction. It is helpful when students study and discuss the recorded lecture with others. The teacher saves time because the lecture is not actually given. The author's experience over the past few years has been positive: the subsequent discussions on the topic with the students have been deeper and have been more applying the theory. Shortly after the first version of this article, the COVID‐19 crisis in the Netherlands caused a complete lockdown of all university activities. All theoretical teaching changed to online teaching. This phenomenon accelerated the development of many online learning methods. Based on the experience during the crisis, the increased use of online learning will probably be more permanent for theoretical education. During the COVID‐19 lockdown, seminar classes have been held online using Microsoft Teams. The first impression of these online seminars indicated that the learning process was more teacher rather learner oriented (https://tauu.uu.nl/docentencommunity/student-centered-learning/). It appeared to be more difficult to have good, interactive discussions. This view supports that face‐to‐face classroom discussions after preparation during self‐study remain an important part of a student‐centred learning process. The necessity of thorough evaluation and further research on the efficiency and success of the different learning methods is evident.

Given that there are growing concerns about the use of living animals for teaching purposes, the author's expectation is that the importance of skills labs will further increase in different veterinary colleges (Dilly, Read, & Baillie, 2017). The use of models for training will grow as more and more mannequins are developed. New techniques, like soft plastination, will enable more realistic models. At FVMU, a model based on soft plastination of a female dog has been made to allow training in vaginal examination (Anonymous, 2019). For welfare reasons, it can be expected that students will first have to demonstrate a minimal level of competency in a model before practicing on a living animal.

Haptic technology, although expensive, is especially interesting in mannequins, as several different programs can be running in one model. The technology provides a fair to good image of reality. As technology increases, the programming of a haptic device might become easier and cheaper. Other haptic programs have been created (Crossan et al., 2001). Even though the number of different situations is still limited, it allows demonstrating less frequently presented pathological situations. Whereas only some students will usually encounter such a patient during their clinical rotations, by using a haptic model all students can practice a situation in addition to obtaining theoretical descriptions. For instance, in bovine obstetrics, the author would like to have a uterine torsion model for diagnosing during a rectal examination, as it is a frequent cause of dystocia (Hirsbrunner & Bühler, 2018; Jonker, Dorresteijn, Roelofsen, & van Werven, 2018).

For practical training of surgical and anaesthetic procedures, in the future mannequins might be paired with computer simulations. In human medicine, these sophisticated but expensive models are already in use. A more affordable model is now in use at Washington State University's College of Veterinary Medicine; it pairs a computer simulation with a simple mannequin and an anaesthetic machine and vital signs monitor (Wuest, 2020). Using programmes like this will enable practicing with less pressure before handling living animals.

Skills laboratory training significantly increases the level of basic technical skills and students’ confidence, even when only relatively simple models are used. However, skills laboratory training, especially with mannequins, takes time because only one student can practice at a time. Using trained peers as trainers might be a valuable solution and a cost‐efficient way of teaching. This approach would save staff time, as expert teachers only have to be present during a relatively short period to assist with specific questions (Herrmann‐Werner et al., 2017). Furthermore, the peers are closer to the students making it easier for them to communicate (Bell, Rhind, Stansbie, & Hudson, 2017; Molgaard & Read, 2017; Riaz, 2014). Similar to other studies, there have been good experiences at FVMU in using trained peers in teaching practical classes.

Preparing e‐learning modules and instruction videos takes a considerable amount of time and money, and thus careful planning is necessary. Personally, the author was not completely aware of what instruction material is already available in universities and on the web. This creates the risk that new material is made while good quality material is already available. A lot of information is also available on YouTube, Google, and Wikipedia and is frequently used (Judd & Elliott, 2017). However, this approach can be risky for learners because only a small proportion of YouTube videos are of sufficient quality (Duncan, Yarwood‐Ross, & Haigh, 2013). Therefore, teachers should provide specific information on which material can be used by students to prepare their skills. At FVMU, e‐learning and videos are made available through the LMS. However, more open access is possible; the college in Hannover uses this approach by placing the instructional videos on a special YouTube channel (Müller et al., 2019). However, in the author's opinion, not all material is suitable for open access, because skills may contain techniques, like foetotomy, that can be easily misunderstood by non‐professionals. This type of content should only be available in a non‐open manner, preferably with institutional access. In the Netherlands, the website medicaleducation.nl is intended for the distribution of medical computer‐based training lessons. Several instructional materials from different Dutch medical colleges are available and can be found and used by students using their institutional access. In Europe, a comparable veterinary site should be developed that can be entered through institutional access. The European Association of Establishments for Veterinary Education (EAEVE) or societies like ESDAR could have a coordination role. The content should preferably be owned by the original makers to facilitate easy updating.

## 9 IN CONCLUSION: THE FUTURE AND LESSONS LEARNED

Changing a course curriculum takes time to produce the new material and to acclimatise learners and teachers to new teaching methods. Time and money (material and staff) must be invested before the profit of better education with fewer contact hours is achieved. Overloading the amount of information in a course should be avoided: less is more. It is essential to determine the minimal level of knowledge of each course or curriculum. E‐learning modules and instructional videos are complementary to textbooks and syllabi; they cannot replace face‐to‐face teaching, practical classes, and the use of teaching animals. Skills laboratories will be more and more important for preparation, but training with live animals or patients will remain necessary. Careful evaluation and further research of the new teaching methods is required. A European‐wide repository on e‐learning material is needed. Last but not least, to create an optimal education preferably early in the curriculum, students should be instructed and learn how to study when using a flipped classroom model with increased self‐study time. Students need to realise their own responsibility in obtaining a sufficient level of knowledge and skills as a starting point to develop their competency. This phenomenon is further extended during lifelong learning.

## CONFLICTS OF INTEREST

The author has no conflicts of interest to declare.

## Data Availability

Data sharing is not applicable to this article as no new data were created or analysed in this review.
